# On Modeling the Earthquake Insurance Data via a New Member of the T-*X* Family

**DOI:** 10.1155/2020/7631495

**Published:** 2020-09-19

**Authors:** Zubair Ahmad, Eisa Mahmoudi, Omid Kharazmi

**Affiliations:** ^1^Department of Statistics, Yazd University, P.O. Box 89175-741, Yazd, Iran; ^2^Department of Statistics, Faculty of Sciences, Vali-e-Asr University of Rafsanjan, Rafsanjan, Iran

## Abstract

Heavy-tailed distributions play an important role in modeling data in actuarial and financial sciences. In this article, a new method is suggested to define new distributions suitable for modeling data with a heavy right tail. The proposed method may be named as the Z-family of distributions. For illustrative purposes, a special submodel of the proposed family, called the Z-Weibull distribution, is considered in detail to model data with a heavy right tail. The method of maximum likelihood estimation is adopted to estimate the model parameters. A brief Monte Carlo simulation study for evaluating the maximum likelihood estimators is done. Furthermore, some actuarial measures such as value at risk and tail value at risk are calculated. A simulation study based on these actuarial measures is also done. An application of the Z-Weibull model to the earthquake insurance data is presented. Based on the analyses, we observed that the proposed distribution can be used quite effectively in modeling heavy-tailed data in insurance sciences and other related fields. Finally, Bayesian analysis and performance of Gibbs sampling for the earthquake data have also been carried out.

## 1. Introduction

In a number of applied areas such as finance and actuarial sciences, data sets are most often positive, and the respective distribution is unimodal hump-shaped and skewed to right having heavier tails as compared to the well-known classical distributions. These distributions are not much flexible to adequately model such types of heavy-tailed data sets. For example, (i) the Pareto distribution, which is frequently used to model financial data sets, does not provide a reasonable fit for many applications, for example, if we are interested in modeling only especially moderate-to-large losses altogether, then in such cases, the Pareto distribution may not be a suitable choice to use [1], and (ii) the Weibull model is capable of catering the behavior of small losses very closely, but, unfortunately, fails to provide an adequate fit to the large losses [2]. In such circumstances, the utilization of the heavy-tailed models may be a good choice to apply. For positive data, heavy-tailed distributions are those whose right-tail probabilities are greater than the exponential one [3], that is,

(1) $$\begin{array}{c} \underset{x⟶\infty }{\text{lim}}e^{\text{px}}\left(1-F\left(x;\xi \right)\right)=\infty ,\;p>0, \end{array}$$

where *F*(*x*; *ξ*) is the cumulative distribution function (cdf) depending on the parameter vector *ξ* ∈ *ℝ*.

Due to the usefulness and flexibility of the heavy-tailed models in financial and actuarial practice, actuaries are always intended to propose new statistical distributions. Therefore, serious attempts have been made to propose new statistical models and are still growing rapidly. The new contribution is made via different approaches such as (i) transformation of variables, (ii) composition of two or more distributions, (iii) compounding of distributions, and (iv) finite mixture of distributions, see Ahmad et al. [4].

Recent investigation of Eling [5] and Adcock et al. [6] determined that skew-normal and skew Student's *t* distributions are the most excellent competitors because the skewed distributions adjust right-skewness and high kurtosis; for the interested readers, one can refer to Shushi [7] and Punzo et al. [8]. However, insurance losses and monetary risks take values on the positive real line, and subsequently, these skew models may not be a suitable choice to use as they are defined on *ℝ*. In such circumstances, the transformation of variable approach, especially, the exponential transformation, has demonstrated to be considerable; for details, see Azzalini et al. [9]. Bagnato and Punzo [10] showed that the transformation method of introducing new distributions is easy to use; however, most often, the inferences become complicated.

Another useful method of proposing new versatile heavy-tailed distributions, which provide the best fit to the heavy-tailed losses, is the methodology of composition, see Paula et al. [11], Klugman et al. [12], Nadarajah and Bakar [13] and Bakar et al. [14]. However, it ought to be noted that the new statistical models introduced via this approach involve more than three parameters inflicting difficulties in estimating the parameters, and more computational efforts are needed.

Another approach of introducing new distributions to cater data modeling adequately with unimodality is compounding of distributions, see Punzo et al. [15] and Mazza and Punzo [16]. Unfortunately, the density function of the distributions obtained by this approach might not have a closed-form expression that makes the estimation more complicated as shown in Punzo et al. [15].

The method of finite mixture models is another prominent approach to obtain new, very flexible models which are able to capture, for example, multimodality of the distribution under consideration, see Bernardi et al. [17], Miljkovic and Grün [18], and Punzo et al. [15]. No doubt, the distributions obtained via this approach are much flexible, but the inferences become more complicated and computationally challenging.

Furthermore, Dutta and Perry [19] performed an empirical analysis of loss distributions, and risk was estimated by different approaches such as exploratory data analysis and other empirical approaches. These authors rejected the idea of using the exponential, gamma, and Weibull models in modeling insurance losses due to the poor results. They concluded that one would need to use a model that is flexible enough in its structure. This motivated the researchers to search for more flexible models offering greater accuracy in fitting the heavy-tailed data.

Hence, bringing flexibility to a model by introducing additional parameter(s) is a desirable feature [20–24]. In a number of recent papers, serious attempts have been made to introduce a variety of new heavy-tailed distributions, see Ahmad et al. [25] and Ahmad et al. [26]. Due to the importance of statistical distributions in financial science, a new family of distributions, called the Z-family, is introduced. The proposed family is introduced via the T*-X* family approach [27]. To illustrate the usefulness of the proposed method, a three-parameter submodel, called the Z-Weibull distribution, is taken and studied in detail. The proposed distribution provides a better description of the earthquake insurance data with possibly heavy tails than the available (i) two-parameter distributions such as Weibull, Burr-XII (B-XII), and generalized exponential (GE), (ii) three-parameter Weibull-claim (W-claim) and exponentiated Lomax (EL), and (iii) four-parameter beta-Weibull (BW) distributions, and possibly many others.

The rest of the article is structured in the following way. The proposed method is introduced in Section 2. The Z-Weibull model is considered in Section 3, and the shapes of its probability density function (pdf) are investigated in the same section. Estimation of parameters is discussed in Section 4. In the same section, a detailed Monte Carlo simulation study is conducted. Actuarial measures of the proposed method along with a simulation study are provided in Section 5. Distribution fit to the earthquake insurance data set is discussed in Section 6. Bayesian analysis as well as Gibbs sampling procedure for the real data set is discussed in Section 7. Future frame work is discussed in Section 8. Finally, some concluding remarks are presented in Section 9.

## 2. Development of the Z-Family

Let *p*(*t*) be the pdf of a random variable, say *T*, where *T* ∈ [*n*_1_, *n*_2_] for −*∞* ≤ *n*_1_, *n*_2_ ≤ *∞*, and let *W*[*F*(*x*; *ξ*)] be a function of *F*(*x*; *ξ*) of a random variable, say *X*, depending on the vector parameter *ξ* satisfying the conditions given in the following:

1. *W*[*F*(*x*; *ξ*)] ∈ [*n*_1_, *n*_2_]
2. *W*[*F*(*x*; *ξ*)] is differentiable and monotonically increasing
3. *W*[*F*(*x*; *ξ*)]⟶*n*_1_ as *x*⟶−*∞* and *W*[*F*(*x*; *ξ*)]⟶*n*_2_ as *x*⟶*∞*

Alzaatreh et al. [27] introduced a general method for generating new families of distributions called the T-*X* family which is defined by

(2) $$\begin{array}{c} G\left(x\right)=\int_{n_{1}}^{W\left[F\left(x;\xi \right)\right]}p\left(t\right)\text{d}t,\;t\geq 0, \end{array}$$

where *W*[*F*(*x*; *ξ*)] satisfies the conditions mentioned above. The probability density function (pdf) corresponding to (2) is given by

(3) $$\begin{array}{c} g\left(x\right)=\left\{\frac{d}{\text{d}x}W\left(F\left(x;\xi \right)\right)\right\}p\left(W\left[F\left(x;\xi \right)\right]\right). \end{array}$$

Deploying the T-*X* proposal, several new classes of distributions have been introduced in the literature [28]. Let *X* have the exponential distribution with the pdf given by

(4) $$\begin{array}{c} p\left(t\right)=\theta e^{-\theta t},\;t,\theta >0. \end{array}$$

Using *θ*=1 in (4), we get

(5) $$\begin{array}{c} p\left(t\right)=e^{-t},\;t>0. \end{array}$$

On setting *W*[*F*(*x*; *ξ*)]=−log{(1 − *F*(*x*; *ξ*))/(*β*^*F*(*x*; *ξ*)^)} and *p*(*t*)=*e*^−*t*^ in (2), we define the cdf of the Z-family of distributions by

(6) $$\begin{array}{c} G\left(x;\beta ,\xi \right)=1-\left\{\frac{1-F\left(x;\xi \right)}{\beta^{F\left(x;\xi \right)}}\right\},\;\beta >0,\;x,\xi \in \mathbb{R}, \end{array}$$

where *F*(*x*; *ξ*) is the baseline cdf. The expression in (6) represents a wide family of univariate continuous distributions. Clearly, when *β*=1, the cdf of the proposed family derived in (6) becomes identical to the baseline cdf. The pdf corresponding to (6) is given by

(7) $$\begin{array}{c} g\left(x;\beta ,\xi \right)=f\left(x;\xi \right)\left(\frac{1+\left(\log\;\beta \right)\left[1-F\left(x;\xi \right)\right]}{\beta^{F\left(x;\xi \right)}}\right),\;x\in \mathbb{R}. \end{array}$$

The survival function (sf) and hazard rate function (hrf) corresponding to (6) are, respectively, given by

(8) $$\begin{array}{c} S\left(x;\beta ,\xi \right)=\frac{1-F\left(x;\xi \right)}{\beta^{F\left(x;\xi \right)}},\;x\in \mathbb{R},\\ h\left(x;\beta ,\xi \right)=f\left(x;\xi \right)\left\{\frac{1+\left(\log\;\beta \right)\left[1-F\left(x;\xi \right)\right]}{1-F\left(x;\xi \right)}\right\},\;x\in \mathbb{R}. \end{array}$$

Due to induction of the extra parameter, the Z-family provides greater distributional flexibility. The key motivations for using the Z-family in the practice are as follows:

- A very useful and simple method of introducing an additional parameter to generalize the existing distributions
- To improve the characteristics and flexibility of the existing models
- To introduce new distributions having closed form of cdf and sf, as well as hrf
- To extend the existing distributions by introducing only one parameter, rather than adding two or more parameters
- To provide the best fit to heavy-tailed insurance data sets
- To provide better fits than other modified models having same or higher number of parameters

## 3. The Z-Weibull Distribution

Most of the extended forms of distributions are introduced for one of the following aims: (i) an extension of the existing model to improve its characteristics, (ii) to obtain new distribution having a heavy right tail, and (iii) to introduce a model whose empirical fit is good to data. Here, we discuss the Z-Weibull distribution that can possess at least one of these aims. The Weibull random variable has the cdf and pdf given by *F*(*x*; *ξ*)=1 − *e*^−*γx*^*α*^^ and *f*(*x*; *ξ*)=*θγx*^*α*−1^*e*^−*γx*^*α*^^, respectively, where *ξ*=(*α*, *γ*). Then, the cdf of the Z-Weibull distribution has the following form:

(9) $$\begin{array}{c} G\left(x;\beta ,\xi \right)=1-\frac{e^{-\gamma x^{\alpha }}}{\beta^{\left(1-e^{-\gamma x^{\alpha }}\right)}},\;x\geq 0,\;\alpha ,\gamma ,\beta >0. \end{array}$$

The corresponding density is given by

(10) $$\begin{array}{c} g\left(x;\beta ,\xi \right)=\frac{\alpha \gamma x^{\alpha -1}e^{-\gamma x^{\alpha }}}{\beta^{\left(1-e^{-\gamma x^{\alpha }}\right)}}\left(1+\left(\log\;\beta \right)e^{-\gamma x^{\alpha }}\right),\;x>0. \end{array}$$

The sf, hrf, and reversed hazard rate function (rhrf) of the proposed model are given by

(11) $$\begin{array}{c} S\left(x;\beta ,\xi \right)=\frac{e^{-\gamma x^{\alpha }}}{\beta^{\left(1-e^{-\gamma x^{\alpha }}\right)}},\;x>0,\\ h\left(x;\beta ,\xi \right)=\alpha \gamma x^{\alpha -1}\left(1+\left(\log\;\beta \right)e^{-\gamma x^{\alpha }}\right),\;x>0,\\ r\left(x;\beta ,\xi \right)=\frac{\alpha \gamma x^{\alpha -1}e^{-\gamma x^{\alpha }}}{\beta^{\left(1-e^{-\gamma x^{\alpha }}\right)}-e^{-\gamma x^{\alpha }}}\left(1+\left(\log\;\beta \right)e^{-\gamma x^{\alpha }}\right),\;x>0, \end{array}$$

respectively.

**Figure 1:**
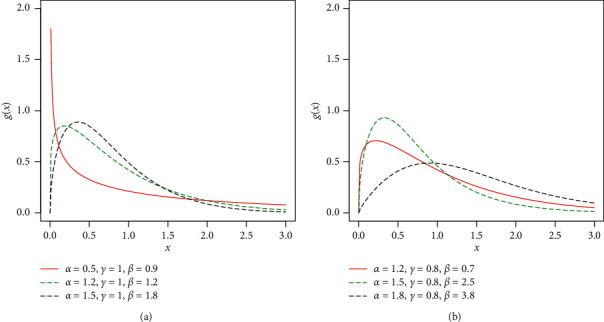
Plots for the density function of the Z-Weibull distribution.

Different plots for the pdf of the Z-Weibull distribution for selected parameter values are given in Figure 1.

## 4. Estimation and Monte Carlo Simulation Study

Several approaches to estimate the model parameter have been introduced in the literature, but the maximum likelihood estimation method is the most commonly employed. The maximum likelihood estimators (MLEs) enjoy several desirable properties and can be used for constructing confidence intervals and regions and also in test statistics. The normal approximation for MLEs in large samples can be easily handled either analytically or numerically. So, we estimate the parameters of the Z-family of distributions from complete samples via the maximum likelihood estimation method. Furthermore, we perform a comprehensive Monte Carlo simulation study to evaluate the performance of the MLEs.

### 4.1. Maximum Likelihood Estimation

In this section, we obtain the MLEs of the model parameters of the Z-family of distributions from complete samples only. Let *x*_1_, *x*_2_,…, *x*_*n*_ be the observed values from the Z-family of distributions with parameters *β* and *ξ*. The total log-likelihood function corresponding to (7) is given by

(12) $$\begin{array}{c} \ell \left(\beta ,\xi \right)=\sum_{i=1}^{n}\log\left[f\left(x_{i};\xi \right)\right]-\sum_{i=1}^{n}F\left(x_{i};\xi \right)\left(\log\;\beta \right)+\sum_{i=1}^{n}\log\left[1+\left(\log\;\beta \right)\left(1-F\left(x_{i};\xi \right)\right)\right]. \end{array}$$

The partial derivatives of (12) are

(13) $$\begin{array}{c} \frac{\partial }{\partial \beta }\ell \left(\beta ,\xi \right)=-\overset{n}{\underset{i=1}{\sum }}\frac{F\left(x_{i};\xi \right)}{\beta }+\frac{1}{\beta }\sum_{i=1}^{n}\frac{\left(1-F\left(x_{i};\xi \right)\right)}{1+\left(\log\;\beta \right)\left(1-F\left(x_{i};\xi \right)\right)},\\ \frac{\partial }{\partial \xi }\ell \left(\beta ,\xi \right)=\overset{n}{\underset{i=1}{\sum }}\frac{\partial f\left(\left(x_{i};\xi \right)/\partial \xi \right)}{f\left(x_{i};\xi \right)}-\overset{n}{\underset{i=1}{\sum }}\frac{\left(\log\;\beta \right)\partial F\left(\left(x_{i};\xi \right)/\partial \xi \right)}{F\left(x_{i};\xi \right)}-\overset{n}{\underset{i=1}{\sum }}\frac{\partial F\left(\left(x_{i};\xi \right)/\partial \xi \right)}{1+\left(\log\;\beta \right)\left(1-F\left(x_{i};\xi \right)\right)}. \end{array}$$

Setting (∂/∂*β*)*ℓ*(*β*, *ξ*) and (∂/∂*ξ*)*ℓ*(*β*, *ξ*) equal to zero and solving numerically these expressions simultaneously yield the MLEs of (*β*, *ξ*).

### 4.2. Monte Carlo Simulation Study

This section offers a comprehensive simulation study to assess the behavior of the MLEs. The Z-family is easily simulated by inverting (6) as follows: if *U* has a uniform *U* (0,1) distribution, then the nonlinear equation is

(14) $$\begin{array}{c} \log\left(1-u\right)+\gamma x^{\alpha }+\left(1-e^{-\gamma x^{\alpha }}\right)\left(\log\;\beta \right)=0. \end{array}$$

**Figure 2:**
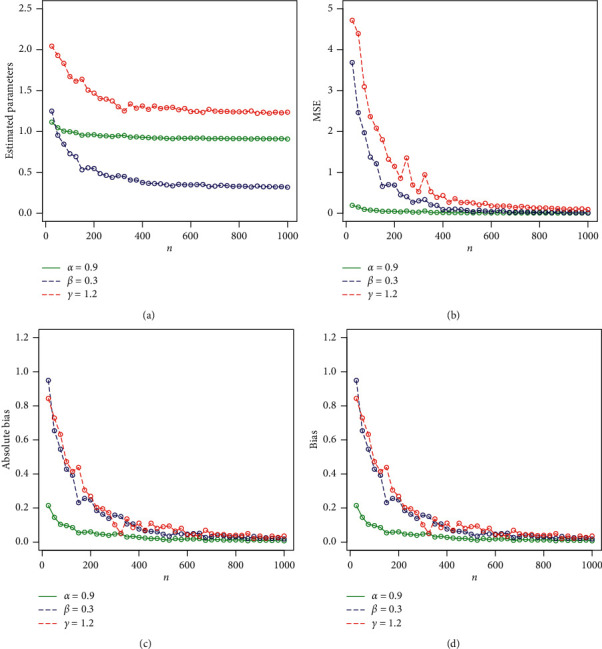
Graphical display of the results provided in Table 1. (a) Plot of estimated parameters vs. *n*. (b) Plot of MSE vs. *n*. (c) Plot of absolute bias vs. *n*. (d) Plot of bias vs. *n*.

**Figure 3:**
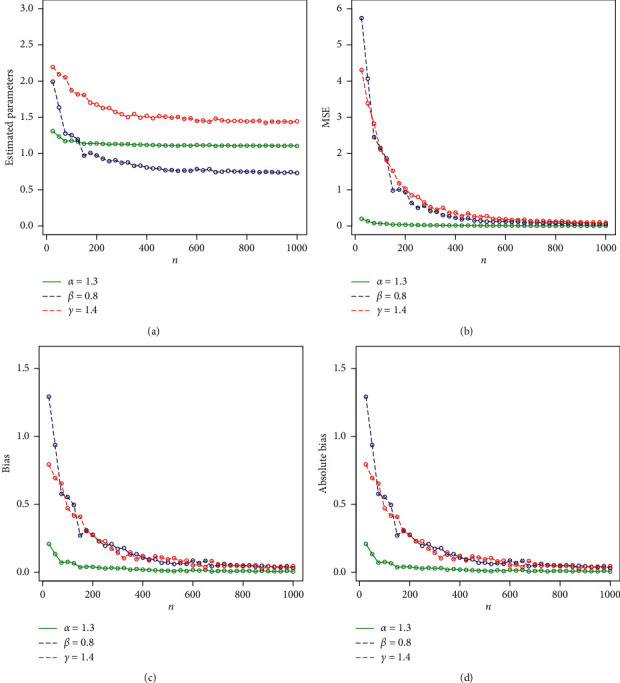
Graphical display of the results provided in Table 2. (a) Plot of estimated parameters vs. *n*. (b) Plot of MSE vs. *n*. (c) Plot of bias vs. *n*. (d) Plot of absolute bias vs. *n*.

Expression (14) can be used to simulate any special subcase of the Z-family. Here, we consider the Z-Weibull distribution to assess the behavior of the MLEs of the proposed method. We simulate the Z-Weibull distribution for two sets of parameters (set 1: *α*=0.9, *β*=0.3, and *γ*=0.5 and set 2: *α*=1.3, *β*=0.8, and *γ*=1). The simulation is performed via statistical software R through the library (rootSolve) command mle. The number of Monte Carlo replications made was 750 times. For maximizing the log-likelihood function, we use the method = “L-BFGS-B” algorithm with optim(). The evaluation of the estimators was performed via the following quantities for each sample size: the empirical mean squared errors (MSEs) are calculated using the R package from the Monte Carlo replications. The MLEs are determined for each piece of simulated data, say, $\left({\hat{\alpha }}_{i},{\hat{\gamma }}_{i},{\hat{\beta }}_{i}\right)$ for *i*=1,2,…, 750, and the biases and MSEs are computed, respectively, by

(15) $$\begin{array}{c} \text{bias}\left(w\right)=\left(\frac{1}{750}\right)\sum_{i=1}^{750}\left({\hat{w}}_{i}-w\right),\\ \text{MSE}\left(w\right)=\left(\frac{1}{750}\right)\sum_{i=1}^{750}{\left({\hat{w}}_{i}-w\right)}^{2}, \end{array}$$

for *w*=*α*, *γ*, *β*. We consider the sample sizes of *n*=25,100,200,300,400,500,600,700,750. The empirical results are given in Tables 1 and 2. Corresponding to Tables 1 and 2, the simulation results are graphically displayed in Figures 2 and 3. Based on Tables 1 and 2 and Figures 2 and 3, the following results are concluded:

- Biases for all parameters are positive
- The parameters tend to be stable
- Estimated biases decrease when the sample size *n* increases
- Estimated MSEs decay toward zero when the sample size *n* increases

## 5. Actuarial Measures

One of the most important tasks of actuarial science institutions is to evaluate the exposure to market risk in a portfolio of instruments, which arise from changes in underlying variables such as prices of equity, interest rates, or exchange rates. In this section, we calculate some important risk measures such as value at risk (VaR) and tail value at risk (TVaR) for the proposed distribution, which play a crucial role in portfolio optimization under uncertainty.

### 5.1. Value at Risk

In the context of actuarial sciences, the VaR is widely used by practitioners as a standard financial market risk measure. It is also known as the quantile risk measure or quantile premium principle. The VaR is always specified with a given degree of confidence, say *q* (typically 90%, 95%, or 99%), and represents the percentage loss in the portfolio value that will be equaled or exceeded only *X* percent of the time. VaR of a random variable *X* is the *q*^th^ quantile of its cdf, see Artzner [29]. If *X* follows the proposed method, then the VaR of *X* is

(16) $$\begin{array}{c} x_{q}=G^{-1}\left(u\right)=F^{-1}\left(t\right), \end{array}$$

where *t* is the solution of *β*^*t*^(1 − *q*)+*t* − 1=0.

### 5.2. Tail Value at Risk

Another important measure is TVaR, also known as conditional tail expectation (CTE) or tail conditional expectation (TCE), which is used to quantify the expected value of the loss given that an event outside a given probability level has occurred. Let *X* follow the Z-Weibull distribution; then, TVaR of *X* is derived as

(17) $$\begin{array}{c} {\text{TVaR}}_{q}\left(x\right)=\left(\frac{1}{1-q}\right)\int_{{\text{VaR}}_{q}}^{\infty }x\text{g}\left(x\right)\text{d}x. \end{array}$$

Using (10) in (17), we get

(18) $$\begin{array}{c} {\text{TVaR}}_{q}\left(x\right)=\left(\frac{\alpha \gamma }{1-q}\right)\int_{{\text{VaR}}_{q}}^{\infty }\frac{x^{\alpha +1-1}e^{-\gamma x^{\alpha }}}{\beta^{\left(1-e^{-\gamma x^{\alpha }}\right)}}\left(1+\left(\log\;\beta \right)e^{-\gamma x^{\alpha }}\right)\text{d}x,\\ {\text{TVaR}}_{q}\left(x\right)=\left(\frac{\alpha \gamma }{1-q}\right)\sum_{i=0}^{\infty }\sum_{j=0}^{i}\frac{{\left(\log\;\beta \right)}^{i}{\left(-1\right)}^{\left(i+j\right)}}{\left(i-j\right)!j!}\int_{{\text{VaR}}_{q}}^{\infty }x^{\alpha +1-1}e^{-\gamma \left(j+1\right)x^{\alpha }}\text{d}x+\left(\frac{\alpha \gamma }{1-q}\right)\sum_{i=0}^{\infty }\sum_{j=0}^{i}\frac{{\left(\log\;\beta \right)}^{i}{\left(-1\right)}^{i+j}}{\left(i-j\right)!j!}\left(\log\;\beta \right)\int_{{\text{VaR}}_{q}}^{\infty }x^{\alpha +1-1}e^{-\gamma \left(j+2\right)x^{\alpha }}\text{d}x. \end{array}$$

Recall the definition of incomplete gamma function in the form Γ(*α*, *x*)=∫_*x*_^*∞*^*t*^*α*−1^*e*^−*t*^d*t*, so from (18), we get

(19) $$\begin{array}{c} {\text{TVaR}}_{q}\left(x\right)=\left(\frac{1}{1-q}\right)\sum_{i=0}^{\infty }\sum_{j=0}^{i}\frac{{\left(\log\;\beta \right)}^{i}{\left(-1\right)}^{\left(i+j\right)}}{\left(i-j\right)!j!\gamma^{\left(1/\alpha \right)}}\frac{\Gamma \left(\left(1/\alpha \right)+1,\gamma \left(j+1\right){\left({\text{VaR}}_{q}\right)}^{\alpha }\right)}{{\left(j+1\right)}^{\left(\left(1/\alpha \right)+1\right)}}+\frac{1}{1-q}\sum_{i=0}^{\infty }\sum_{j=0}^{i}\frac{{\left(\log\;\beta \right)}^{i}{\left(-1\right)}^{\left(i+j\right)}}{\left(i-j\right)!j!\gamma^{\left(1/\alpha \right)}}\frac{\Gamma \left(\left(1/\alpha \right)+1,\gamma \left(j+2\right){\left({\text{VaR}}_{q}\right)}^{\alpha }\right)}{{\left(j+2\right)}^{\left(\left(1/\alpha \right)+1\right)}}. \end{array}$$

### 5.3. Numerical Study of the Risk Measures

n this section, we provide numerical study of the VaR and TVaR for the Weibull and Z-Weibull distributions for different sets of parameters. The process is described as follows:

1. Random samples of size *n* = 100 are generated from the Weibull and Z-Weibull models
2. The parameters have been estimated via the maximum likelihood method
3. 1000 repetitions are made to calculate the VaR and TVaR

**Figure 4:**
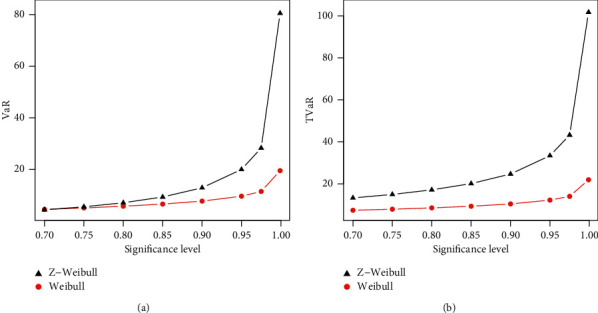
Graphical sketching of the results of the VaR and TVaR provided in Table 3.

**Figure 5:**
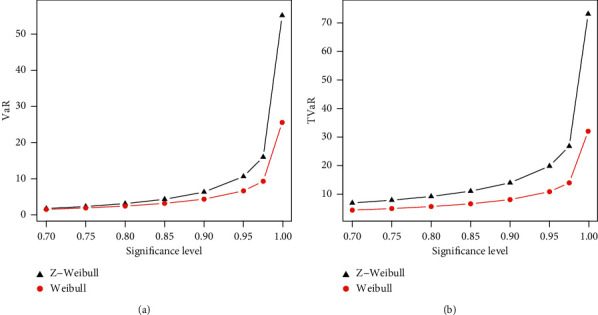
Graphical sketching of the results of the VaR and TVaR provided in Table 4.

The numerical results of the risk measures are provided in Tables 3 and 4 and displayed graphically in Figures 4 and 5 corresponding to each table.

The simulation is performed for Weibull and Z-Weibull for the selected values of parameters. A model with higher values of the risk measures is said to have a heavier tail. The simulated results provided in Tables 3 and 4 show that the proposed Z-Weibull model has higher values of the risk measures than the traditional Weibull distribution. The simulation results are graphically displayed in Figures 4 and 5, which show that the proposed model has a heavier tail than the Weibull distribution.

## 6. Practical Illustration via the Earthquake Insurance Data

The main applications of the heavy tail models are the so-called extreme value theory or insurance loss phenomena. In this section, we consider heavy-tailed earthquake insurance data to illustrate the usefulness of the Z-Weibull model. The data are reported by the “National Centers for Environmental Information” available at https://ngdc.noaa.gov/hazard/earthqk.shtml. We compare the goodness-of-fit results of the Z-Weibull distribution with the other well-known heavy-tailed distributions. The distribution functions of the competitive models are as follows:

(i) W-claim:
  (20) $$\begin{array}{c} G\left(x\right)=\frac{\sigma {\left(1-e^{-\gamma x^{\alpha }}\right)}^{2}}{1-\left(1-\sigma \right)\left(1-e^{-\gamma x^{\alpha }}\right)},\;x\geq ,\alpha ,\gamma ,\sigma >0. \end{array}$$

(ii) Weibull:

(21) $$\begin{array}{c} G\left(x\right)=1-e^{-\gamma x^{\alpha }},\;x\geq 0,\alpha ,\gamma >0. \end{array}$$

(iii) B-XII:
  (22) $$\begin{array}{c} G\left(x\right)=1-{\left(1+x^{c}\right)}^{-k},\;x\geq 0,c,k>0. \end{array}$$

(iv) GE:
  (23) $$\begin{array}{c} G\left(x\right)={\left(1-e^{-\gamma x^{\alpha }}\right)}^{a},\;x\geq 0,a,\alpha ,\gamma >0. \end{array}$$

(v) EL:
  (24) $$\begin{array}{c} G\left(x\right)={\left(1-{\left(1+\gamma x\right)}^{-\alpha }\right)}^{a},\;x\geq 0,a,\alpha ,\gamma >0. \end{array}$$

(vi) BW:
  (25) $$\begin{array}{c} G\left(x\right)=I_{\left(1-e^{-\gamma x^{\alpha }}\right)}\left(a,b\right),\;x\geq 0,a,b,\alpha ,\gamma >0. \end{array}$$

Next, we consider certain analytical measures in order to verify which distribution fits better the considered data. These analytical measures include (i) discrimination measures, such as Akaike information criterion (AIC), Bayesian information criterion (BIC), Hannan–Quinn information criterion (HQIC), and consistent Akaike information criterion (CAIC), and (ii) two other goodness-of-fit measures including Anderson–Darling (AD) test statistic and −2*ℓ*. The discrimination measures are given as follows.
(vii) Akaike information criterion is given by
  (26) $$\begin{array}{c} \text{AIC}=2k-2\ell . \end{array}$$

(viii) Consistent Akaike information criterion is given by
  (27) $$\begin{array}{c} \text{CAIC}=\frac{2\text{nk}}{n-k-1}-2\ell . \end{array}$$

(ix) Bayesian information criterion is given by
  (28) $$\begin{array}{c} \text{BIC}=k\;\log\left(n\right)-2\ell . \end{array}$$

(x) Hannan–Quinn information criterion is given by

(29) $$\begin{array}{c} \text{HQIC}=2k\;\log\left(\log\left(n\right)\right)-2\ell , \end{array}$$

  where *ℓ* denotes the log-likelihood function, *k* is the number of model parameters, and *n* is the sample size.
(xi) The AD test statistic is given by

(30) $$\begin{array}{c} \text{AD}=-n-\left(\frac{1}{n}\right)\sum_{i=1}^{n}\left(2i-1\right)\left[\log\;G\left(x_{i}\right)+\log\left\{1-G\left(x_{n-i+1}\right)\right\}\right], \end{array}$$

where *n* is the sample size and *x*_*i*_ is the *i*^th^ observation in the sample, calculated when the data are sorted in the ascending order.

All the computations are carried out using the optim() R-function with the argument method = “BFGS” (see Appendix). A model with lowest values for these measures could be chosen as the best model to fit the data. The values of MLEs of the parameters along with standard errors in parenthesis are presented in Table 5, whereas the discrimination measures are displayed in Table 6. The AD statistic and −2*ℓ* are provided in Table 7. Based on the considered data set, we have observed that the Z-Weibull distribution is the best fitted model among the above considered models.

**Figure 6:**
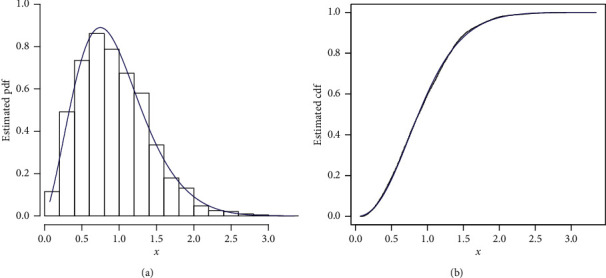
Estimated pdf and cdf of the Z-Weibull distribution.

**Figure 7:**
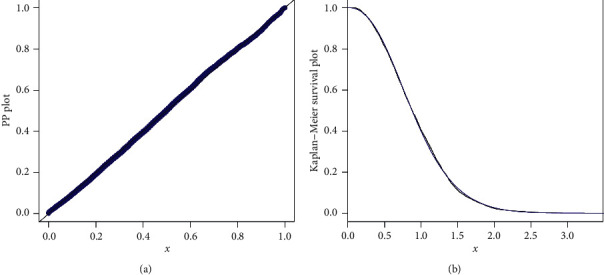
PP and Kaplan–Meier survival plots of the Z-Weibull distribution.

**Figure 8:**
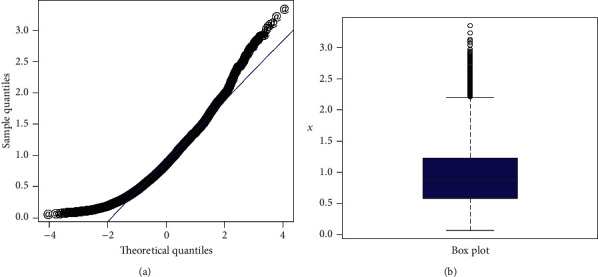
QQ plot of the Z-Weibull distribution and box plot of the earthquake insurance data.

As we can see, the results show that the Z-Weibull distribution provides a better fit than the other competitors. Hence, the proposed model can be used as a best candidate model for modeling insurance data sets. Furthermore, in support of Tables 6 and 7, the estimated cdf and pdf are plotted in Figure 6. The Kaplan–Meier survival plot and PP plot are sketched in Figure 7, whereas the QQ plot of the proposed distribution and box plot of the earthquake data are presented in Figure 8. From the estimated pdf in Figure 6, it is clear that the proposed distribution provides an adequate fit to the heavy-tailed earthquake data. From Figures 6 and 7, we can easily detect that the proposed distribution fits the estimated cdf and Kaplan–Meier survival plots very closely. The PP and QQ plots which serve as a tool for graphical display of the analytical measures show that the Z-Weibull distribution provides the best fit to real data. Finally, the box plot (Figure 8) of the data is graphical evidence that the data possess tail skewed to the right.

Furthermore, using the earthquake insurance data, we obtained the values of the Kolmogorov–Smirnov (KS) statistic of the proposed and other competing models. Then, we applied the parametric bootstrap technique [30] and bootstrapped the *p* value for all the competing models. The KS statistic and the corresponding bootstrapped *p* value are provided in Table 8. Based on the results provided in Table 8, we conclude that the proposed model is the best candidate model among the competing distributions for modeling the insurance claim data.

## 7. Bayesian Estimation

Bayesian inference procedure has been taken into consideration by many statistical researchers, especially those in the field of survival analysis and reliability engineering. In this section, complete sample data are analyzed through the Bayesian point of view. We assume that the parameters *α*, *γ*, and *β* of the Z-Weibull distribution have independent prior distributions as follows:

(31) $$\begin{array}{c} \alpha ∼\text{Gamma}\left(a,b\right),\\ \gamma ∼\text{Gamma}\left(c,d\right),\\ \beta ∼\text{Gamma}\left(e,f\right), \end{array}$$

where *a*, *b*, *c*, *d*, *e*, and *f* are positive. More about choosing gamma priors, refer Kundu and Howlader [31], S. Dey and T. Dey [32], Dey et al. [33], and Dey et al. [34]. Hence, the joint prior density function is formulated as follows:

(32) $$\begin{array}{c} \pi \left(\alpha ,\gamma ,\beta \right)=\frac{b^{\alpha }d^{c}f^{e}}{\left(\Gamma a\right)\left(\Gamma c\right)\left(\Gamma e\right)}\alpha^{a-1}\gamma^{c-1}\gamma^{e-1}\exp\left\{-\left(b\alpha +d\gamma +f\beta \right)\right\}. \end{array}$$

In the Bayesian estimation, the actual value of the parameter may be adversely affected by the loss when choosing an estimator. This loss can be measured by a function of the parameter and the corresponding estimator. Five well-known loss functions and associated Bayesian estimators and corresponding posterior risk are presented in Table 9.

For more details, see Calabria and Pulcini [35] and Dey et al. [36]. Next, we provide the posterior probability distribution for a complete data set. We define the function *φ* as

(33) $$\begin{array}{c} \varphi \left(\alpha ,\gamma ,\beta \right)=\alpha^{a-1}\gamma^{c-1}\beta^{e-1}\exp\left\{-\left(b\alpha +d\gamma +f\beta \right)\right\},\;\alpha >0,\;\gamma >0,\;\beta >0,\;\beta \neq 1. \end{array}$$

The joint posterior distribution in terms of a given likelihood function *L*(data) and joint prior distribution *π*(*α*, *γ*, *β*) is defined as

(34) $$\begin{array}{c} \pi^{*}\left(\left.\alpha ,\gamma ,\beta \right|\text{data}\right)\alpha \pi \left(\alpha ,\gamma ,\beta \right)L\left(\text{data}\right). \end{array}$$

Hence, the joint posterior density of parameters *α*, *γ*, and *β* for complete sample data is obtained by combining the likelihood function and joint prior density (32). Therefore, the joint posterior density function is given by

(35) $$\begin{array}{c} \pi^{*}\left(\left.\alpha ,\gamma ,\beta \right|\underline{x}\right)=K\varphi \left(\left.\alpha ,\gamma ,\beta \right|\underline{x}\right)\overset{n}{\underset{i=1}{\prod }}\frac{\alpha \gamma x_{i}^{\alpha -1}e^{-\gamma x_{i}^{\alpha }}\left(1+\log\left(\beta \right)\right)e^{-\gamma x_{i}^{\alpha }}}{\beta^{1-e^{-\gamma x_{i}^{\alpha }}}}, \end{array}$$

where

(36) $$\begin{array}{c} K^{-1}=\int_{0}^{\infty }\int_{0}^{\infty }\int_{0}^{\infty }\varphi \left(\alpha ,\gamma ,\beta \right)\overset{n}{\underset{i=1}{\prod }}\frac{\alpha \gamma x_{i}^{\alpha -1}e^{-\gamma x_{i}^{\alpha }}\left(1+\log\left(\beta \right)\right)e^{-\gamma x_{i}^{\alpha }}}{\beta^{1-e^{-\gamma x_{i}^{\alpha }}}}\text{d}\alpha \text{d}\gamma \text{d}\beta . \end{array}$$

Moreover, the marginal posterior density of *α*, *γ*, and *β*, assuming that Θ=(*α*, *γ*, *β*), is given by

(37) $$\begin{array}{c} \pi \left(\left.\Theta_{i}\right|\underline{x}\right)=\int_{0}^{\infty }\int_{0}^{\infty }\pi^{*}\left(\left.\Theta_{i}\right|\underline{x}\right)\text{d}\Theta_{j}\text{d}\Theta_{k}, \end{array}$$

where *i*, *j*, *k*=1,2,3, *i* ≠ *j* ≠ *k*, and also Θ_*i*_ is the *i*-th member of vector Θ.

From (35) and (37), it is clear that there is no closed form for the Bayesian estimators under the five loss functions described in Table 8. Therefore, we use the MCMC procedure based on 10,000 replicates to compute Bayesian estimators.

Because of intractable integrals associated with joint posterior and marginal posterior distributions, one needs to use numerical software to solve the integral equations numerically. The two most popular MCMC methods are the Metropolis–Hastings algorithm [37, 38] and the Gibbs sampling [39]. Gibbs sampling is a special case of the Metropolis–Hastings algorithm which generates a Markov chain by sampling from the full set of conditional distributions. Often, Bayesian inference requires computing intractable integrals to generate posterior samples. In practice, simulations related to Gibbs sampling are conducted through special software WinBUGS. WinBUGS software was developed in 1997 to simulate data of complex posterior distributions, where analytical or numerical integration techniques cannot be applied. One may also use OpenBUGS software, which is an open-source version of WinBUGS. Using Gibbs sampling, we obtain samples from the joint posterior distribution and then use OpenBUGS software to carry out the Bayesian analysis.

The process is described as follows:

- Gibbs sampling technique is used to generate posterior samples
- 10,000 replicates are made to compute the Bayesian estimators via OpenBUGS software
- The idea of Congdon [40] was to implement and choose *a*=*b*=*c*=*d*=*e*=*f*=0.0001 as we do not have any prior information about hyperparameters

**Figure 9:**
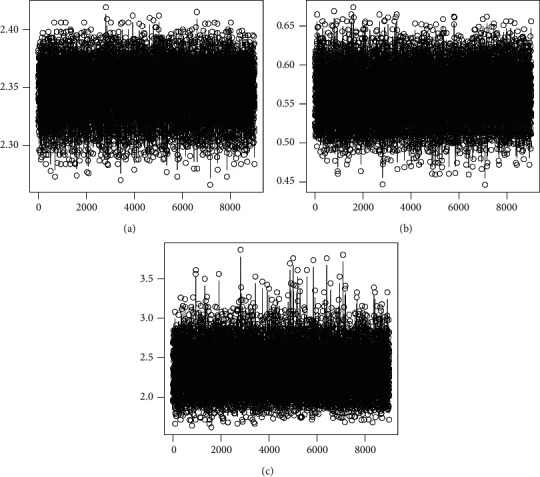
Trace plots of each parameter of the Z-Weibull distribution. (a) Alpha. (b) Gamma. (c) Beta.

**Figure 10:**
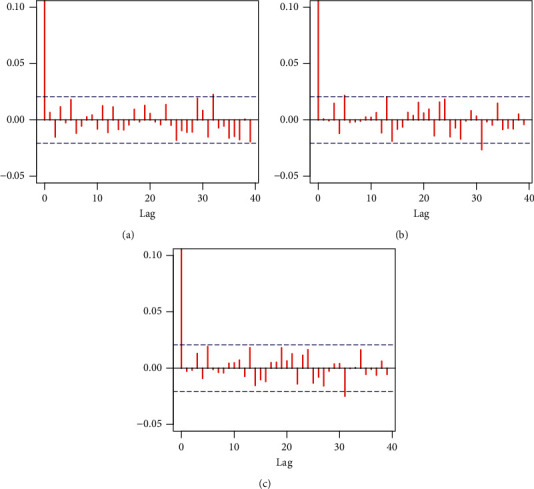
Autocorrelation plots of each parameter of the Z-Weibull distribution. (a) Alpha. (b) Gamma. (c) Beta.

**Figure 11:**
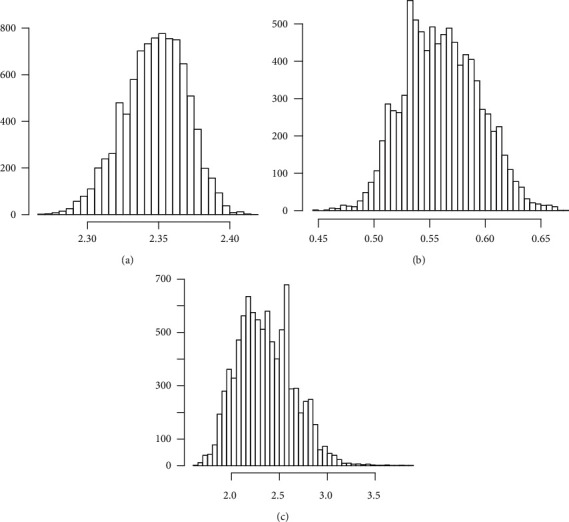
Histogram plots of each parameter of the Z-Weibull distribution. (a) Alpha. (b) Gamma. (c) Beta.

The corresponding Bayesian estimates and posterior risk are provided in Table 10.  Table 11 provides 95% credible and HPD intervals for each parameter of the Z-Weibull distribution. Moreover, we provide the posterior summary plots in Figures 9–11. These plots confirm that the sampling process is of the prime quality, and the convergence does occur.

## 8. Discussion and Future Framework

Statistical decision theory addresses the state of uncertainty and provides a rational framework for dealing with problems of actuarial and financial decision-making. The insurance data sets are generally skewed to the right and heavy-tailed. The traditional distributions are not flexible enough to counter complex forms of data such as insurance science data.

Due to the importance of statistical distributions in actuarial sciences, a number of papers have been appeared in the literature aiming to improve the characteristics of the existing distributions. Although this has been achieved, unfortunately, the numbers of parameters have been increased, and the estimation of parameters and derivation of mathematical problems become complicated.

To provide a better description of the insurance science data, therefore, in this study, an attempt has been made to introduce a new family of statistical distributions aiming to increase the flexibility of the existing distributions. A special submodel of the proposed family offers the best fitting to the heavy-tailed insurance science data. The maximum likelihood method is adopted to estimate the model parameters, and a comprehensive Monte Carlo simulation study is done to evaluate the behavior of the estimators.

To show the usefulness of the proposed method in insurance sciences, a real-life application of the earthquake insurance data is discussed. Analyzing the data set, it showed that the proposed model performs much better than the other competitive distributions.

From the above discussion, it is obvious that the researchers are always in search of new flexible distributions. Therefore, to bring further flexibility in the proposed model, we suggest to introduce its extended versions. The proposed method can further be extended by introducing a shape parameter to the model.

(i) A random variable *X* is said to follow the extended version of the Z-family if its cdf is given by
  (38) $$\begin{array}{c} G\left(x;a,\beta ,\xi \right)=1-\left\{\frac{1-F{\left(x;\xi \right)}^{a}}{\beta^{F\left(x;\xi \right)}}\right\},\;a,\beta >0,x,\xi \in \mathbb{R}, \end{array}$$

where *a* is the additional shape parameter. For *a*=1, expression (38) reduces to (6). The new proposal may be named as the exponentiated Z-family. For the illustrative purposes, one may consider its special subcase, called the exponentiated Z-Weibull (EZ-Weibull) distribution, defined by the cdf
  (39) $$\begin{array}{c} G\left(x;a,\beta ,\xi \right)=1-\left\{\frac{1-{\left(1-e^{-\gamma x^{\alpha }}\right)}^{a}}{\beta^{\left(1-e^{-\gamma x^{\alpha }}\right)}}\right\},\;a,\alpha ,\gamma ,\beta >0,x\in \mathbb{R}. \end{array}$$

Due to the introduction of the additional shape parameter, the suggested extension may be much flexible in modeling data in insurance sciences and other related fields.
(ii) Another extension of the Z-family is given by
  (40) $$\begin{array}{c} G\left(x;\theta ,\beta ,\xi \right)=1-{\left\{\frac{1-F\left(x;\xi \right)}{\beta^{F\left(x;\xi \right)}}\right\}}^{\theta },\;\theta ,\beta >0,x,\xi \in \mathbb{R}, \end{array}$$

  where *θ*  is the additional shape parameter. For *θ*=1, expression (40) reduces to (6). The model defined in (40) may be named as the extended Z-family.
(iii) Another generalized version of the Z-family can be introduced via

(41) $$\begin{array}{c} G\left(x;a,\theta ,\beta ,\xi \right)=1-{\left\{\frac{1-F{\left(x;\xi \right)}^{a}}{\beta^{F\left(x;\xi \right)}}\right\}}^{\theta },\;a,\theta ,\beta >0,x,\xi \in \mathbb{R}, \end{array}$$

where *a* and *θ*  are the additional shape parameters. Clearly, for *a*=1, expression (41) reduces to (40), and for *θ*=1, expression (41) reduces to (38). However, for *a*=*θ*=1, expression (41) reduces to (6). The model introduced in (41) may be named as the extended exponentiated Z-family.
(iv) Another generalized version of the new extended Z-family can be introduced via
  (42) $$\begin{array}{c} G\left(x;a,\theta ,\beta ,\xi \right)=1-{\left\{\frac{1-F{\left(x;\xi \right)}^{a}}{\beta^{F\left(x;\xi \right)}}\right\}}^{\theta },\;a,\theta ,\beta >0,x,\xi \in \mathbb{R}. \end{array}$$

## 9. Concluding Remarks

A variety of methods for proposing new heavy-tailed distributions have been developed to model data related to financial and actuarial sciences. We carried out this area of research further and introduced a new heavy-tailed distribution family. Some distributional properties are derived, and the method of maximum likelihood estimation is discussed to estimate the model parameters. In addition to distributional properties, some actuarial properties are also derived. Based on the actuarial measures, a comprehensive simulation study is conducted. We focused our concentration on a three-parameter special model called the Z-Weibull distribution. To prove the potential and usefulness of the Z-Weibull distribution, earthquake insurance data are analyzed, and its comparison is made with the other well-known distributions. While analyzing the earthquake insurance data, it is observed that the proposed model performs better than the other competitive models. Bayesian analysis using the earthquake data is also provided. Finally, some new extensions are also suggested which may further improve the characteristics of the proposed family.

## Figures and Tables

**Table 1:** Simulation results for the Z-Weibull distribution.

Set 1: *α*=0.9, *β*=0.3, and *γ*=1.2				
*n*	Parameters	MLEs	MSEs	Bias
25	*α*	1.1150	0.1937	0.2150
	*β*	1.2490	3.6872	0.9490
	*γ*	2.0431	4.7184	0.8431

50	*α*	1.0456	0.1526	0.1456
	*β*	0.9536	2.4614	0.6536
	*γ*	1.9282	4.3933	0.7281

100	*α*	0.9970	0.0783	0.0970
	*β*	0.7277	1.3741	0.4277
	*γ*	1.6722	2.3642	0.4722

200	*α*	0.9605	0.0472	0.0605
	*β*	0.5478	0.6878	0.2478
	*γ*	1.4691	1.1474	0.2691

400	*α*	0.9281	0.0163	0.0281
	*β*	0.3769	0.0901	0.0769
	*γ*	1.3115	0.4336	0.1115

600	*α*	0.9193	0.0085	0.0193
	*β*	0.3471	0.0383	0.0471
	*γ*	1.2424	0.1812	0.0424

800	*α*	0.9128	0.0067	0.0128
	*β*	0.3313	0.0279	0.0313
	*γ*	1.2405	0.1318	0.0405

1000	*α*	0.9006	0.0042	0.0076
	*β*	0.3184	0.0119	0.0184
	*γ*	1.2160	0.0928	0.0360

**Table 2:** Simulation results for the Z-Weibull distribution.

Set 2: *α*=1.3, *β*=0.8, and *γ*=1.4				
*n*	Parameters	MLEs	MSEs	Bias
25	*α*	1.3094	0.2002	0.2094
	*β*	1.9925	5.7399	1.2925
	*γ*	2.1937	4.3061	0.7937

50	*α*	1.2340	0.1333	0.1340
	*β*	1.6368	4.0681	0.9368
	*γ*	2.0939	3.3865	0.6939

100	*α*	1.1766	0.0697	0.0766
	*β*	1.2543	2.1560	0.5543
	*γ*	1.8715	2.1136	0.4715

200	*α*	1.1396	0.0368	0.0396
	*β*	0.9740	0.9313	0.2740
	*γ*	1.6771	1.0320	0.2771

400	*α*	1.1194	0.0153	0.0194
	*β*	0.8080	0.2293	0.1080
	*γ*	1.5204	0.3718	0.1204

600	*α*	1.1171	0.0094	0.0171
	*β*	0.7860	0.1229	0.0860
	*γ*	1.4516	0.1869	0.0516

800	*α*	1.1093	0.0065	0.0093
	*β*	0.7495	0.0833	0.0495
	*γ*	1.4443	0.1219	0.0443

1000	*α*	1.1050	0.0049	0.0050
	*β*	0.7807	0.0537	0.0307
	*γ*	1.4147	0.0935	0.0347

**Table 3:** Simulation results of the VaR and TVaR for the selected values of the parameters.

Dist.	Par	Level of significance	VaR	TVaR
Weibull	*α*=1.2 *γ*=0.85	0.700	4.5373	7.3911
		0.750	5.0945	7.9076
		0.800	5.7601	8.5304
		0.850	6.5967	9.3205
		0.900	7.7437	10.4136
		0.950	9.6399	12.2384
		0.990	11.4765	14.0197
		0.999	19.5352	21.9150

Z-Weibull	*α*=1.2 *β*=1.4 *γ*=0.85	0.700	4.3778	13.2772
		0.750	5.5376	14.9465
		0.800	7.1015	17.1137
		0.850	9.3408	20.1026
		0.900	12.8999	24.6727
		0.950	20.0017	33.4043
		0.990	28.2956	43.2302
		0.999	80.4988	101.7184

**Table 4:** Simulation results of the VaR and TVaR for the selected values of the parameters.

Dist.	Par	Level of significance	VaR	TVaR
Weibull	*α*=0.65 *γ*=0.7	0.700	1.5273	4.4325
		0.750	1.9174	4.9762
		0.800	2.4392	5.6792
		0.850	3.1801	6.6436
		0.900	4.3463	8.1092
		0.950	6.6444	10.8853
		0.990	9.2950	13.9806
		0.999	25.5667	32.0408

Z-Weibull	*α*=0.65 *β*=0.8 *γ*=0.7	0.700	1.7585	6.9318
		0.750	2.3222	7.9131
		0.800	3.1171	9.2175
		0.850	4.3112	11.0668
		0.900	6.3169	13.9926
		0.950	10.6148	19.8560
		0.990	16.0027	26.8019
		0.999	55.1566	73.1187

**Table 5:** Estimated values of the proposed and other competitive models.

Dist.	$\hat{\alpha }$	$\hat{\gamma }$	$\hat{\beta }$	$\hat{a}$	$\hat{b}$	$\hat{c}$	$\hat{k}$	$\hat{\sigma }$
Z-Weibull	2.346 (0.020)	0.564 (0.029)	2.295 (0.248)					
W-claim	2.023 (0.076)	0.709 (0.056)						1.783 (0.197)
Weibull	2.110 (0.091)	0.889 (0.086)						
B-XII						1.480 (0.030)	2.851 (0.107)	
GE		2.283 (0.015)		4.308 (0.053)				
EL	4.804 (4.910)	3.879 (5.400)		4.335 (0.054)				
BW	1.609 (0.055)	0.644 (0.304)		1.616 (0.087)	2.237 (1.228)			

**Table 6:** Discrimination measures of the proposed and other competitive models.

Dist.	AIC	BIC	CAIC	HQIC
Z-Weibull	23286.95	23310.57	23289.92	23294.69
W-claim	23345.86	23364.09	23355.04	23354.73
Weibull	23379.64	23395.38	23386.84	23384.80
B-XII	24197.63	24213.37	24197.63	24202.78
GE	23846.71	23862.45	23853.57	23851.86
EL	23863.99	23887.60	23871.99	23871.72
BW	23338.24	23369.73	23343.17	23348.56

**Table 7:** Analytical measures of the fitted models.

Dist.	−2*ℓ*	AD
Z-Weibull	11640.48	0.840
W-claim	11667.98	0.986
Weibull	11687.82	1.071
B-XII	12096.81	5.139
GE	11921.35	4.965
EL	11928.99	5.012
BW	11665.12	0.952

**Table 8:** KS and the corresponding bootstrapped *p* value of the proposed and other models.

Dist.	KS	Bootstrapped *p* value
Z-Weibull	0.205	0.976
W-claim	0.367	0.806
Weibull	0.476	0.704
B-XII	0.954	0.408
GE	0.590	0.605
EL	0.869	0.502
BW	0.406	0.775

**Table 9:** Bayes estimator and posterior risk under different loss functions.

Loss function	Bayes estimator	Posterior risk
*L*_1_ = SELF = (*θ* − *d*)^2^	*E*(*θ*/*x*)	*V*(*θ*/*x*)
*L*_2_ = WSELF = (((*θ* − *d*)^2^)/*θ*)	(*E*(*θ*^−1^/*x*))^−1^	*E*(*θ*/*x*) − (*E*(*θ*^−1^/*x*))^−1^
*L*_3_ = MSELF = (1 − (*d*/*θ*))^2^	(*E*(*θ*^−1^/*x*))/(*E*(*θ*^−2^/*x*))	1 − ((*E*(*θ*^−1^/*x*)^2^)/(*E*(*θ*^−2^/*x*)^2^))
*L*_4_ = PLF = (((*θ* − *d*)^2^)/*d*)	$\sqrt{E\left(\theta^{2}/x\right)}$	$2\left(\sqrt{E\left(\theta^{2}/x\right)}-E\left(\theta /x\right)\right)$
*L*_5_ = KLF = $\left(\sqrt{\left(d/\theta \right)-\sqrt{\left(d/\theta \right)}}\right)$	$\sqrt{\left(E\left(\theta /x\right)\right)/\left(E\left(\theta^{-1}/x\right)\right)}$	$2\left(\sqrt{E\left(\theta /x\right)E\left(\theta^{-1}/x\right)}-1\right)$

**Table 10:** Bayesian estimates and their posterior risks of the parameters under different loss functions based on the earthquake insurance data.

Bayes	$\hat{\alpha }$		$\hat{\gamma }$		$\hat{\beta }$	
Loss functions	Estimate	Risk	Estimate	Risk	Estimate	Risk
SELF	2.34808	0.00048	0.56078	0.00113	2.35742	0.08661
WSELF	2.34787	0.00020	0.55876	0.00201	2.32162	0.03580
MSELF	2.34766	8.90*e* − 05	0.55675	0.00359	2.28686	0.01497
PLF	2.34819	0.00020	0.56179	0.00202	2.37572	0.03659
KLF	2.34798	8.879*e* − 05	0.55977	0.00360	2.33945	0.01536

**Table 11:** Credible and HPD intervals of the parameters for the earthquake insurance data.

Parameters	Credible interval	HPD interval
*α*	(2.334, 2.364)	(2.303, 2.388)
*γ*	(0.536, 0.585)	(0.410, 0.625)
*β*	(2.139, 2.562)	(1.827, 2.883)

## Data Availability

This work is mainly a methodological development and has been applied on secondary data related to the earthquake insurance data, but if required, data will be provided.

## Appendix

### R-Code for Analysis

Note: in the following R-code, *a* is used for *α*, *g* is used for *γ*, *b* is used for *β*, and pm is used for the proposed model.

  data ← read.csv(file.choose(), header = TRUE)
  data = data[1]
  data = data[!is.na(data)]
  data = data/100
  data
  hist(data)
  ###—Probability density function
  pdf_pm ← function(par,*x*)
  {
*a* = par[1]
*g* = par[2]
*b* = par[3]
  (*a* ∗ *g* ∗ (*x*^(*a* − 1)) ∗ (exp(−*g* ∗ *x*^*a*)) ∗ (*b*^(exp(−*g* ∗ *x*^*a*) − 1))) ∗ (1 + log(*b*) ∗ exp(−*g* ∗ *x*^*a*))
  ###—Cumulative distribution function.
  cdf_pm ← function(par,*x*)
  {
*a* = par[1]
*g* = par[2]
*b* = par[3]
  1 − (exp(−*g* ∗ *x*^*a*)/(*b*^(1 − exp(−*g* ∗ *x*^*a*))))
  }
  set.seed(0)
  goodness.fit(pdf = pdf_pm,
  cdf = cdf_pm,
  starts = *c*(0.5,0.5,0.5), data = data,
  method = “BFGS,” domain = *c*(0,Inf),mle = NULL)
  %%%%---------------------------------------------------
  % Estimated pdf
  %%%%---------------------------------------------------
*x* ← read.csv(file.choose(), header = TRUE)
*x* = *x*[,1]
*x* = *x*[!is.na(*x*)]
*x* = *x*/100
*x*
  ###—Parameter values
*a* = 2.346
*g* = 0.564
*b* = 2.295
  pdf = (*a* ∗ *g* ∗ (*x*^(*a* − 1)) ∗ (exp(−*g* ∗ *x*^*a*)) ∗ (*b*^(exp(−*g* ∗ *x*^*a*) − 1))) ∗ (1 + log(*b*) ∗ exp(−*g* ∗ *x*^*a*))
*f* = pdf
*x* = sort(*x*)
  yrange = *c*(0,1)
  xrange = *c*(min(*x*),max(*x*))
  hist(*x*, freq = FALSE, breaks = 15, xlim = xrange, ylim = yrange,
*y*lab = “Estimated pdf,”xlab = “*x*,” main = ””)
  par(new = TRUE)
  lines(*x*,*f*, xlim = xrange, lty = 1, ylab = ,” ylim = yrange, lwd = 3,
  col = “blue,” xlab = ””)
  par(new = TRUE)
  %%%%---------------------------------------------------
  %Estimated cdf
  %%%%---------------------------------------------------
*x* ← read.csv(file.choose(), header = TRUE)
*x* = *x*[,1]
*x* = *x*[!is.na(*x*)]
*x* = *x*/100
*x*
  ###—Parameter values
*a* = 2.346
*g* = 0.564
*b* = 2.295
*x* ← sort(*x*)
  F1 ← ecdf(*x*)
  ecdf ← F1(*c*(x))
  zweibullcdf ← 1 − ((exp(−*g* ∗ *x*^*a*)/(*b*^(1 − exp(−*g* ∗ *x*^*a*)))))
  plot(*x*, ecdf, lty = 1, lwd = 2.5, type = “*s*,” xlab = “*x*,”
  ylab = “Estimated cdf,” ylim = *c*(0,1), xlim = *c*(min(*x*),
  max(*x*)), col = “black”)
  par(new = TRUE)
  plot(*x*, zweibullcdf, lty = 1, lwd = 3, type = “l,” xlab = “*x*,”
  ylab = “Estimated cdf,” ylim = *c*(0,1), xlim = *c*(min(*x*),
  max(*x*)), col = “blue”)
  par(new = TRUE)
  %%%%---------------------------------------------------
  %Kaplan-Meier survival plot
  %%%%---------------------------------------------------
*x* ← read.csv(file.choose(), header = TRUE)
*x* = *x*[,1]
*x* = *x*[!is.na(*x*)]
*x* = *x*/100
*x*
  ###—Parameter values
  library(survival)
  delta = rep(1,length(*x*))
*x* ← sort(*x*)
  km = survfit(Surv(*x*,delta)∼1)
  plot(km,conf.int = FALSE, ylab = “Kaplan-Meier survival plot,”xlab = “*x*”)
*a* = 2.346
*g* = 0.564
*b* = 2.295
  ss ← function(*x*)
  {
  (exp(−*g* ∗ *x*^*a*)/(*b*^(1 − exp(−*g* ∗ *x*^*a*))))
  }
  lines(seq(0, 3.5, length.out = 100),ss(seq(0, 3.5, length.out = 100)),
  col = “blue,” lwd = 3)
  %%%%---------------------------------------------------
  %PP plot
  %%%%---------------------------------------------------
*x* ← read.csv(file.choose(), header = TRUE)
*x* = *x*[,1]
*x* = *x*[!is.na(*x*)]
*x* = *x*/100
*x*
  cdfLD1 = function(*x*, *a*, *g*, *b*)
  {
  1 − (exp(−*g* ∗ *x*^*a*)/(*b*^(1 − exp(−*g* ∗ *x*^*a*))))
  }
*x* = sort(*x*)
*n* = length(*x*)
  ###—Empirical distribution function
  Fn = seq(1,*n*)/*n*
  plot(Fn, cdfLD1(*x*, 2.346, 0.564, 2.295), xlab = “*x*,”
  ylab = “PP Plot,” pch = 21, col = “blue,” bg = “blue”)
  abline(0,1)
  %%%%---------------------------------------------------
  %QQ plot
  %%%%---------------------------------------------------
*x* ← read.csv(file.choose(), header = TRUE)
*x* = *x*[,1]
*x* = *x*[!is.na(*x*)]
*x* = *x*/100
*x*
  ###—Parameter values
*a* = 2.346
*g* = 0.564
*b* = 2.295
*x* ← sort(*x*)
  F1 ← ecdf(*x*)
  ecdf ← F1(*c*(*x*))
  zweibullcdf ← 1 − (exp(−*g* ∗ *x*^*a*)/(*b*^(1 − exp(−*g* ∗ *x*^*a*))))
  qqnorm(*x*, pch = “@,” col = “black,” main = ””)
  qqline(*x*, pch = “@,” col = “blue,” ylab = “*X*,” lty = 1, lwd = 3)
  %%%%---------------------------------------------------
  %Box plot
  %%%%---------------------------------------------------
*x* < read.csv(file.choose(), header = TRUE)
*x* = *x*[,1]
*x* = *x*[!is.na(*x*)]
*x* = *x*/100
*x*
  boxplot(*x*, main = ,” col = “blue,” ylab = “x,” xlab = “Box Plot”)
  %%%%---------------------------------------------------

## References

[1] Guillen M., Prieto F., Sarabia J. M. (2011). Modelling losses and locating the tail with the Pareto positive stable distribution. *Insurance: Mathematics and Economics*.

[2] Bhati D., Ravi S. (2018). On generalized log-Moyal distribution: a new heavy tailed size distribution. *Insurance: Mathematics and Economics*.

[3] Beirlant J., Matthys G., Dierckx G. (2001). Heavy-tailed distributions and rating. *ASTIN Bulletin*.

[4] Ahmad Z., Mahmoudi E., Hamedani G. G., Kharazmi O. (2020). New methods to define heavy-tailed distributions with applications to insurance data. *Journal of Taibah University for Science*.

[5] Eling M. (2012). Fitting insurance claims to skewed distributions: are the skew-normal and skew-student good models?. *Insurance: Mathematics and Economics*.

[6] Adcock C., Eling M., Loperfido N. (2015). Skewed distributions in finance and actuarial science: a review. *The European Journal of Finance*.

[7] Shushi T. (2017). Skew-elliptical distributions with applications in risk theory. *European Actuarial Journal*.

[8] Punzo A., Mazza A., Maruotti A. (2018). Fitting insurance and economic data with outliers: a flexible approach based on finite mixtures of contaminated gamma distributions. *Journal of Applied Statistics*.

[9] Azzalini A., Del Cappello T., Kotz S. (2002). Log-skew-normal and log-skew-*t* distributions as models for family income data. *Journal of Income Distribution*.

[10] Bagnato L., Punzo A. (2013). Finite mixtures of unimodal beta and gamma densities and the *k*-bumps algorithm. *Computational Statistics*.

[11] Paula G. A., Leiva V., Barros M., Liu S. (2012). Robust statistical modeling using the Birnbaum-Saunders-*t* distribution applied to insurance. *Applied Stochastic Models in Business and Industry*.

[12] Klugman S. A., Panjer H. H., Willmot G. E. (2012). *Loss Models: from Data to Decisions*.

[13] Nadarajah S., Bakar S. A. A. (2014). New composite models for the Danish fire insurance data. *Scandinavian Actuarial Journal*.

[14] Bakar S. A., Hamzah N. A., Maghsoudi M., Nadarajah S. (2015). Modeling loss data using composite models. *Insurance: Mathematics and Economics*.

[15] Punzo A., Bagnato L., Maruotti A. (2018). Compound unimodal distributions for insurance losses. *Insurance: Mathematics and Economics*.

[16] Mazza A., Punzo A. (2019). Modeling household income with contaminated unimodal distributions. *New Statistical Developments in Data Science*.

[17] Bernardi M., Maruotti A., Petrella L. (2012). Skew mixture models for loss distributions: a Bayesian approach. *Insurance: Mathematics and Economics*.

[18] Miljkovic T., Grün B. (2016). Modeling loss data using mixtures of distributions. *Insurance: Mathematics and Economics*.

[19] Dutta K., Perry J. (2006). A tale of tails: an empirical analysis of loss distribution models for estimating operational risk capital.

[20] Nasiru S. (2018). Extended odd Fréchet-G family of distributions. *Journal of Probability and Statistics*.

[21] Cortés M. A., Elal-Olivero D., Olivares-Pacheco J. F. (2018). A new class of distributions generated by the extended bimodal-normal distribution. *Journal of Probability and Statistics*.

[22] Al-Mofleh H. (2018). On generating a new family of distributions using the tangent function. *Pakistan Journal of Statistics and Operation Research*.

[23] Mead M. E., Cordeiro G. M., Afify A. Z., Mofleh H. A. (2019). The alpha power transformation family: properties and applications. *Pakistan Journal of Statistics and Operation Research*.

[24] He W., Ahmad Z., Afify A. Z., Goual H. (2020). The arcsine exponentiated-*X* family: validation and insurance application. *Complexity*.

[25] Ahmad Z., Mahmoudi E., Dey S., Khosa S. K. (2020). Modeling vehicle insurance loss data using a new member of T-X family of distribution. *Journal of Statistical Theory & Practice*.

[26] Ahmad Z., Mahmoudi E., Dey S. (2020). A new family of heavy-tailed distribution with an application to the heavy-tailed insurance loss data. *Communication in Statistics: Simulation & Computation*.

[27] Alzaatreh A., Lee C., Famoye F. (2013). A new method for generating families of continuous distributions. *Metron*.

[28] Ahmad Z., Hamedani G. G., Butt N. S. (2019). Recent developments in distribution theory: a brief survey and some new generalized classes of distributions. *Pakistan Journal of Statistics and Operation Research*.

[29] Artzner P. (1999). Application of coherent risk measures to capital requirements in insurance. *North American Actuarial Journal*.

[30] Stute W., Manteiga W. G., Quindimil M. P. (1993). Bootstrap based goodness-of-fit-tests. *Metrika*.

[31] Kundu D., Howlader H. (2010). Bayesian inference and prediction of the inverse Weibull distribution for type-II censored data. *Computational Statistics & Data Analysis*.

[32] Dey S., Dey T. (2014). On progressively censored generalized inverted exponential distribution. *Journal of Applied Statistics*.

[33] Dey S., Ali S., Park C. (2015). Weighted exponential distribution: properties and different methods of estimation. *Journal of Statistical Computation and Simulation*.

[34] Dey S., Dey T., Ali S., Mulekar M. S. (2016). Two-parameter Maxwell distribution: properties and different methods of estimation. *Journal of Statistical Theory and Practice*.

[35] Calabria R., Pulcini G. (1996). Point estimation under asymmetric loss functions for left truncated exponential samples. *Communications in Statistics-Theory and Methods*.

[36] Dey S., Singh S., Tripathi Y. M., Asgharzadeh A. (2016). Estimation and prediction for a progressively censored generalized inverted exponential distribution. *Statistical Methodology*.

[37] Metropolis N., Rosenbluth A. W., Rosenbluth M. N., Teller A. H., Teller E. (1953). Equation of state calculations by fast computing machines. *The Journal of Chemical Physics*.

[38] Hastings W. K. (1970). Monte Carlo sampling methods using Markov chains and their applications. *Biometrika*.

[39] Geman S., Geman D. (1984). Stochastic relaxation, Gibbs distributions, and the Bayesian restoration of images. *IEEE Transactions on Pattern Analysis and Machine Intelligence*.

[40] Congdon P. (2001). *Bayesian Statistical Modelling*.

